# Influence of poor sleep on cardiovascular disease-free life expectancy: a multi-resource-based population cohort study

**DOI:** 10.1186/s12916-023-02732-x

**Published:** 2023-03-02

**Authors:** Bo-Huei Huang, Borja del Pozo Cruz, Armando Teixeira-Pinto, Peter A. Cistulli, Emmanuel Stamatakis

**Affiliations:** 1 Charles Perkins Centre, School of Health Sciences, Faculty of Medicine and Health, the University of Sydney, Camperdown, Australia; 2 Centre for Active and Healthy Ageing, Department of Sports Science and Clinical Biomechanics, University of Southern Denmark, Odense, Denmark; 3 Sydney School of Public Health, Faculty of Medicine and Health, the University of Sydney, Camperdown, Australia; 4 Charles Perkins Centre, Northern Clinical School, Faculty of Medicine and Health, University of Sydney, Sydney, Australia

**Keywords:** Sleep, Life expectancy, Cardiovascular disease, Primary care

## Abstract

**Background:**

The complexity of sleep hinders the formulation of sleep guidelines. Recent studies suggest that different unhealthy sleep characteristics jointly increase the risks for cardiovascular disease (CVD). This study aimed to estimate the differences in CVD-free life expectancy between people with different sleep profiles.

**Methods:**

We included 308,683 middle-aged adults from the UK Biobank among whom 140,181 had primary care data linkage. We used an established composite sleep score comprising self-reported chronotype, duration, insomnia complaints, snoring, and daytime sleepiness to derive three sleep categories: poor, intermediate, and healthy. We also identified three clinical sleep disorders captured by primary care and inpatient records within 2 years before enrollment in the cohort: insomnia, sleep-related breathing disorders, and other sleep disorders. We estimated sex-specific CVD-free life expectancy with three-state Markov models conditioning on survival at age 40 across different sleep profiles and clinical disorders.

**Results:**

We observed a gradual loss in CVD-free life expectancy toward poor sleep such as, compared with healthy sleepers, poor sleepers lost 1.80 [95% CI 0.96–2.75] and 2.31 [1.46–3.29] CVD-free years in females and males, respectively, while intermediate sleepers lost 0.48 [0.41–0.55] and 0.55 [0.49–0.61] years. Among men, those with clinical insomnia or sleep-related breathing disorders lost CVD-free life by 3.84 [0.61–8.59] or 6.73 [5.31–8.48] years, respectively. Among women, sleep-related breathing disorders or other sleep disorders were associated with 7.32 [5.33–10.34] or 1.43 [0.20–3.29] years lost, respectively.

**Conclusions:**

Both self-reported and doctor-diagnosed poor sleep are negatively associated with CVD-free life, especially pronounced in participants with sleep-related breathing disorders.

**Supplementary Information:**

The online version contains supplementary material available at 10.1186/s12916-023-02732-x.

## Background

The World Health Organization has recognized sleep as a critical health state and health-related behavior [1]. Although one-fourth of Europeans have insomnia [2], with very few exceptions [3], there is very little national guidance on sleep, reflecting the complexity of sleep health as a multi-dimensional behavior [4] as well as the lack of longitudinal evidence base on population-wide effects of poor sleep [3, 5].

One major difficulty in evaluating the health impacts of sleep is its complexity as a biological process and behavior [4]. Current estimates of optimal nocturnal sleep range in adults is seven to less than nine hours per night [6, 7], with little evidence-based guidance on other health-related sleep characteristics, such as quality and timing [5, 8]. While most cohorts fail to capture the multi-dimensional nature of sleep, recent studies have highlighted joint effects of different sleep characteristics with risks for cardiovascular disease (CVD) [8, 9]. With a composite sleep score comprising chronotype, duration, insomnia complaint, snoring, and daytime sleepiness, Fan et al. found that healthy sleepers had a 35% lower risk of incident CVD than poor sleepers [9]. Using the same set of sleep measures, our previous work further suggested that poor sleepers had a 39% higher risk of CVD mortality than healthy sleepers [8]. These findings indicated that a traditional mutually adjusted approach could not capture the multiplicative effects of different sleep characteristics, and the derived individual/independent effect of a single characteristic could be spurious.

Compared to ratio-based estimation, years of life lost free of diseases, the difference in disease-free life expectancy between people with different characteristics provides a tangible metric on the burdens of exposures taking both morbidity and mortality into account. For example, CVD-free life expectancy estimates, at a given age, how many years a person would live without CVD until death. Life expectancy is easy to communicate to policymakers, media, and the general population [10–12]. However, no study has used such measures to evaluate the health burden of comprehensive sleep characteristics.

To our knowledge, no prior sleep research has used primary care diagnoses to estimate the health burden of the compromised life expectancy of sleep disorders, although primary care (general practice) records have distinct advantages in capturing incident sleep disorders more comprehensively and objectively [5]. Therefore, this study aimed to estimate CVD-free years of life lost among people with poor sleep, evaluated by self-report and clinical primary care diagnosis.

## Methods

### Data source

We retrieved individual data from the UK Biobank, a population-based prospective cohort that recruited participants in 22 assessment centers throughout England, Wales, and Scotland between 2006 and 2010 (https://www.ukbiobank.ac.uk/). Full cohort details can be found elsewhere [13, 14]. In brief, more than half a million participants (response rate 6%) aged 40 to 69 provided informed consent to enrolment and data linkage to electronic health records. The baseline assessment included self-reported questionnaires, a computer-assisted personal interview, physical examinations, and biological sampling. The UK Biobank keeps removing all records of dropping out participants, so retrieving loss of follow-up is inapplicable. By the time we conducted the current study (October 2021), there were 502,459 participants with data available. The National Health Service and the National Research Ethics Service have approved the UK Biobank (Ref. 11/NW/0382).

### Electronic health records

The UK Biobank repository accesses national registered data on hospital inpatient diagnoses and death for all participants across the UK through the National Health Service (NHS). The NHS encoded records with the International Classification of Diseases (ICD), of which both the Ninth Revision (ICD-9) and the Tenth Revision (ICD-10) were available for the inpatient records, while only ICD-10 was applied to the death registry. Inpatient records were availed from 1981 onwards. Both registries were censored up to March 2021, with slight differences between countries (Additional file 1: Method S1).

Primary care data on clinical events and prescriptions covered 227,834 participants. Primary care clinical events were coded with Read codes version 2 (Read v2) or Clinical Terms Version 3 (CTV3), while prescriptions were mainly coded with Read v2, British National Formulary (BNF), or Dictionary of Medicines and Devices (dm + d). Further details of primary care data sources, cleaning, and harmonization are provided in Additional file 1: Method S2 and Fig. S1-2. The primary care was censored to September 2018, with data availability varied between participants and was determined by the registration date with a primary care practice.

### CVD incidence and all-cause mortality

We identified the incidence of hospital inpatient disease events based on ICD-9 and ICD-10: CVD (I01 to I09, I11, I13, I21 to I25, I27, I3, I4, I50, I51, I6, I7, I80 to I84, I87 to I9, R54) [10, 15]. The corresponding ICD-9 was provided in Additional file 1: Table S1. The date of events and death were retrieved from the NHS electronic health records described above.

### Sleep Exposure

As part of the baseline questionnaire, participants reported five sleep characteristics, based on which we further identified five healthy phenotypes, including no usual insomnia complaints, adequate sleep duration (7 to < 9 h/day), no snoring, morning chronotype, and no frequent daytime sleepiness [9]. We scored participants from 0 to 5, according to the count of healthy characteristics and categorized them into three groups: “healthy sleep” (≥ 4 composite sleep score); “intermediate sleep” (2 or 3 score); and “poor sleep” (≤ 1 score). This categorization has been proven to distinguish different CVD risk profiles, and the simple addition approach showed a similar predictive power to a weighted score [9]. The original questions and options were provided in Additional file 1: Table S2. These definitions and scores have shown excellent convergent validity with CVD incidence and mortality [8, 9].

We identified recent clinical sleep disorder events (2 years before enrolment) based on inpatient admissions, primary care clinical events, and sleep disorder-specific prescriptions (BNF Chapter 4 Sect. 1: hypnotics and anxiolytics). Modified from the definition provided by the American Sleep Association and American Academy of Sleep Medicine [16, 17], we distinguished five different sleep disorders, including insomnia, hypersomnia, sleep-related breathing disorders, circadian rhythm sleep disorders, and parasomnias (including sleep-related bruxism). In addition, we grouped hypersomnia, circadian rhythm sleep disorders, parasomnias, non-specific sleep disorders (e.g., “poor sleep pattern”), and sleep medication prescriptions without a corresponding clinical event into “other sleep disorders.” We provided detailed codes for each classification system in Additional file 1: Table S3-5. Since the clinical diagnosis of sleep disorders mainly refers to more than one self-reported sleep characteristic, we did not further integrate both self-reported and clinically unhealthy sleep.

### Statistical analysis

We set the follow-up from the UK Biobank enrollment to the date of death, inpatient or death registry censoring, or the 81^st^ birthday, whichever came first. To avoid overestimation, we constrained the censoring age (τ restriction) at 81, corresponding to the oldest age of death in the analyzed sample [18]. Considering the pathophysiological differences in CVD risks between sexes [19], we stratified all our models by sex. To evaluate the sex-specific CVD-free residual lifetime of participants with different exposures, we applied continuous-time three-state Cox Markov survival models [20, 21]. All participants started with the CVD-free state and subsequently either (a) maintained healthy until censored, (b) were diagnosed with the disease(s) and lived until censored, (c) maintained healthy then deceased, or (d) were diagnosed with the disease(s) then deceased. We estimated state-specific residual lifetime at age 40 [22], and calculated the total and CVD-free years of life lost by comparing the marginal life expectancies spent in a healthy state between the population with questionnaire-based poor/intermediate sleep (exposure) and those with healthy sleep (reference), or between the population with each diagnosed sleep disorder (exposure) and those without (reference). Based on a priori defined directed acyclic graphs (Additional file 1: Fig. S3) [23], we selected the following potential covariates: age, socioeconomic status, mental health issues, perceived health, body mass index (BMI), economic activity, and shift work, cigarette smoking, alcohol consumption, diet quality, discretionary screen time, and physical activity. The detailed definitions and the original questions of covariates were provided in Additional file 1: Table S6. Models examining each self-reported sleep characteristic or diagnosed sleep disorder were further mutually adjusted for the remaining characteristics or disorders. For all the life expectancy estimations, we applied median/ mean value for categorical/numerous covariates, and for estimations of each self-reported sleep characteristic/clinical sleep disorder, the remaining characteristics or disorders were set to healthy.

We obtained the 95% CIs for life expectancy estimations via nonparametric bootstrapping with 1000 iterations. All data were processed in SAS 9.4 (on secured local computers), and analyses were performed on R 4.1.1 (on secured local computers) or R 4.0.4 (on the University of Sydney’s high-performance computing cluster Artemis). To improve comparability, we also estimated hazard ratios (HR) with Fine-Gray sub-distribution hazards models, in which non-CVD death was entered as a competing risk to incident CVD [24]. Covariates stayed the same as in the primary analyses.

## Results

**Fig. 1:**
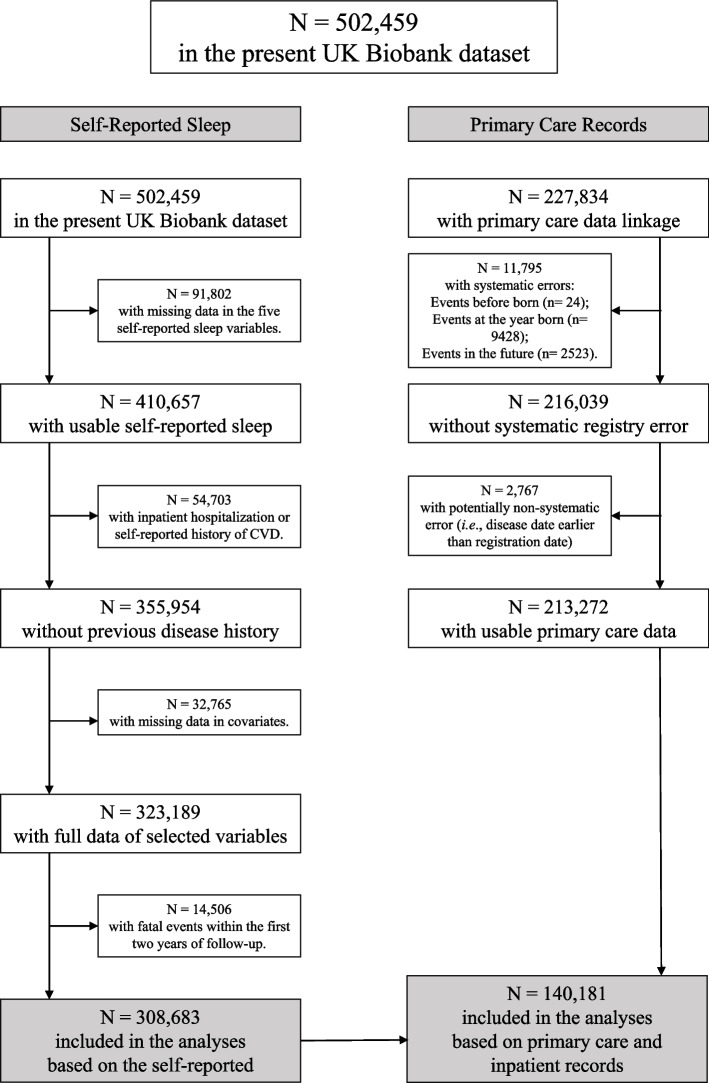
The participants’ exclusion flowchart. Abbreviations: CVD, cardiovascular diseases. The detailed missing pattern was provided in Additional file 1: Table S7

We sequentially excluded participants with missing data in any of the five self-reported sleep characteristics (*n* = 91,802), with an inpatient or self-reported history of CVD (*n* = 54,703), with missing covariates (*n* = 32,765), and with incident CVD or fatal events within the first 2 years of follow-up (*n* = 14,506), leaving 308,683 participants for the core analysis of self-reported and 140,181 participants for the clinically confirmed sleep events data (Fig. 1, Additional file 1: Table S7). Among the 308,683 participants, with a mean follow-up of 11.8 (1.3) years from a mean age 56.2 (8.1), there were 13,790 deaths from any cause and 53,064 incident CVD, corresponding to 3,653,450 person-years at risk of death (Table 1). At baseline, 60% of the participants reported healthy sleep, followed by 38% and 2% reporting intermediate and poor sleep, respectively (Table 1, Additional file 1: Table S8). Participants who suffered from mental health problems, perceived poor health, consumed a low-quality diet, had high BMI, faced higher socioeconomic deprivation, retired, smoked/drank more, spent more discretionary time on screen, and exercised less tended to have poor sleep in both sexes. Women were more likely to report healthy sleep despite no appreciable differences in corresponding diagnoses (Additional file 1: Table S9).

**Table 1:** The basic characteristics of participants with different questionnaire-based sleep characteristics

Variables	Females				Males				All
	Poor	Intermediate	Healthy	Total	Poor	Intermediate	Healthy	Total	
n	3407 (2)	61,966 (36)	108,173 (62)	173,546	3072 (2)	55,408 (41)	76,657 (57)	135,137	308,683
Early Chronotype	244 (7)	26,097 (42)	83,570 (77)	109,911 (63)	272 (9)	21,666 (39)	61,522 (80)	83,460 (62)	193,371 (63)
Adequate Duration (7 to < 9 h/day)	107 (3)	25,030 (40)	96,644 (89)	121,781 (70)	142 (5)	25,696 (46)	68,307 (89)	94,145 (70)	215,926 (70)
No Usual Insomnia Complaints	120 (4)	24,898 (40)	96,648 (89)	121,666 (70)	179 (6)	34,074 (61)	71,636 (93)	105,889 (78)	227,555 (74)
No Snoring	219 (6)	32,929 (53)	92,525 (86)	125,673 (72)	153 (5)	16,488 (30)	53,623 (70)	70,264 (52)	195,937 (63)
No Frequent Daytime Sleepiness	2534 (74)	59,611 (96)	107,709 (100)	169,854 (98)	2119 (69)	53,252 (96)	76,375 (100)	131,746 (97)	301,600 (98)
Follow-up Time (yr)	11.8 (1.3)	11.8 (1.2)	11.9 (1.1)	11.9 (1.2)	11.7 (1.5)	11.8 (1.4)	11.8 (1.3)	11.8 (1.4)	11.8 (1.3)
Incident CVD	714 (21)	9973 (16)	14,264 (13)	24,951 (14)	854 (28)	12,161 (22)	15,098 (20)	28,113 (21)	53,064 (17)
All-Cause Death	180 (5)	2396 (4)	3476 (3)	6052 (3)	213 (7)	3380 (6)	4145 (5)	7738 (6)	13,790 (4)
Age of Enrollment	56.6 (7.4)	56.6 (7.7)	55.8 (8.2)	56.1 (8.0)	55.7 (8.0)	56.1 (8.2)	56.3 (8.2)	56.2 (8.2)	56.2 (8.1)
Socioeconomic Status									
Highest Deprivation	1062 (31)	14,579 (24)	22,137 (20)	37,778 (22)	925 (30)	13,109 (24)	15,874 (21)	29,908 (22)	67,686 (22)
2nd Deprivation	853 (25)	15,910 (26)	27,294 (25)	44,057 (25)	795 (26)	13,619 (25)	18,868 (25)	33,282 (25)	77,339 (25)
3rd Deprivation	780 (23)	15,732 (25)	28,919 (27)	45,431 (26)	684 (22)	14,159 (26)	20,287 (26)	35,130 (26)	80,561 (26)
Least Deprivation	712 (21)	15,745 (25)	29,823 (28)	46,280 (27)	668 (22)	14,521 (26)	21,628 (28)	36,817 (27)	83,097 (27)
Mental Health Issues	2000 (59)	28,506 (46)	38,360 (35)	68,866 (40)	1286 (42)	15,619 (28)	16,710 (22)	33,615 (25)	102,481 (33)
Perceived Health									
Excellent	248 (7)	8917 (14)	25,133 (23)	34,298 (20)	235 (8)	7938 (14)	16,906 (22)	25,079 (19)	59,377 (19)
Good	1556 (46)	36,941 (60)	68,436 (63)	106,933 (62)	1337 (44)	32,088 (58)	46,949 (61)	80,374 (59)	18,7307 (61)
Fair	1138 (33)	13,631 (22)	13,320 (12)	28,089 (16)	1063 (35)	13,132 (24)	11,692 (15)	25,887 (19)	53,976 (17)
Poor	465 (14)	2477 (4)	1284 (1)	4226 (2)	437 (14)	2250 (4)	1110 (1)	3797 (3)	8023 (3)
Body Mass Index (kg/m2)									
< 30	1986 (58)	45,184 (73)	89,543 (83)	136,713 (79)	1877 (61)	40,272 (73)	61,634 (80)	103,783 (77)	240,496 (78)
30 to < 40	1190 (35)	14,968 (24)	17,226 (16)	33,384 (19)	1084 (35)	14,339 (26)	14,474 (19)	29,897 (22)	63,281 (21)
> = 40	231 (7)	1814 (3)	1404 (1)	3449 (2)	111 (4)	797 (1)	549 (1)	1457 (1)	4906 (2)
Economic Activity and Shift Work									
Retired/not in the workforce	1651 (48)	26,566 (43)	42,105 (39)	70,322 (41)	1180 (38)	18,159 (33)	24,779 (32)	44,118 (33)	114,440 (37)
Employed not in shift work	1417 (42)	29,820 (48)	57,847 (53)	89,084 (51)	1381 (45)	29,616 (53)	43,483 (57)	74,480 (55)	163,564 (53)
Employed in night shift work	166 (5)	2501 (4)	3307 (3)	5974 (3)	325 (11)	4577 (8)	4505 (6)	9407 (7)	15,381 (5)
Employed in day shift work	173 (5)	3079 (5)	4914 (5)	8166 (5)	186 (6)	3056 (6)	3890 (5)	7132 (5)	15,298 (5)
Cigarette Smoking									
Never	1671 (49)	34,819 (56)	68,510 (63)	105,000 (61)	1266 (41)	26,288 (47)	41,967 (55)	69,521 (51)	174,521 (57)
Cessation	1189 (35)	20,647 (33)	32,600 (30)	54,436 (31)	1278 (42)	21,370 (39)	27,170 (35)	49,818 (37)	104,254 (34)
Current	547 (16)	6500 (10)	7063 (7)	14,110 (8)	528 (17)	7750 (14)	7520 (10)	15,798 (12)	29,908 (10)
Alcohol Drinking									
Never	170 (5)	2892 (5)	5170 (5)	8232 (5)	64 (2)	1114 (2)	1791 (2)	2969 (2)	11,201 (4)
Quit	175 (5)	2059 (3)	3051 (3)	5285 (3)	137 (4)	1522 (3)	2177 (3)	3836 (3)	9121 (3)
< 14 UK Units/wk	2178 (64)	40,306 (65)	74,036 (68)	116,520 (67)	1175 (38)	21,933 (40)	33,350 (44)	56,458 (42)	172,978 (56)
14 to < 28 UK Units/wk	553 (16)	11,667 (19)	19,728 (18)	31,948 (18)	687 (22)	14,793 (27)	21,668 (28)	37,148 (27)	69,096 (22)
> = 28 UK Units/wk	331 (10)	5042 (8)	6188 (6)	11,561 (7)	1009 (33)	16,046 (29)	17,671 (23)	34,726 (26)	46,287 (15)
Dietary Quality									
Poor	226 (7)	2535 (4)	3034 (3)	5795 (3)	491 (16)	6921 (12)	6730 (9)	14,142 (10)	19,937 (6)
Intermediate	2082 (61)	36,714 (59)	61,282 (57)	100,078 (58)	2041 (66)	36,785 (66)	50,107 (65)	88,933 (66)	189,011 (61)
Healthy	1099 (32)	22,717 (37)	43,857 (41)	67,673 (39)	540 (18)	11,702 (21)	19,820 (26)	32,062 (24)	99,735 (32)
Discretionary Screen Time (h/day)	4.5 (2.6)	3.8 (2.0)	3.4 (1.8)	3.6 (1.9)	4.7 (2.6)	4.2 (2.2)	3.8 (2.0)	4.0 (2.1)	3.8 (2.0)
Physical Activity									
No MVPA	850 (25)	11,686 (19)	15,716 (15)	28,252 (16)	729 (24)	8942 (16)	9287 (12)	18,958 (14)	47,210 (15)
< 10 MET h/week	470 (14)	7131 (12)	10,440 (10)	18,041 (10)	382 (12)	6531 (12)	7354 (10)	14,267 (11)	32,308 (10)
10 to < 20 MET h/week	469 (14)	9779 (16)	17,268 (16)	27,516 (16)	427 (14)	8314 (15)	11,144 (15)	19,885 (15)	47,401 (15)
> = 20 MET hrs/wk	1618 (47)	33,370 (54)	64,749 (60)	99,737 (57)	1534 (50)	31,621 (57)	48,872 (64)	82,027 (61)	181,764 (59)

Data were shown in *N* (% vertically) or mean (SD) for categorical or continuous variables, respectively

A summary without sex stratification was provided in Additional file 1: Table S8 and a characteristic table of participants with different diagnosed sleep disorders was provided in Additional file 1: Table S9

*Abbreviations*: *CVD* Cardiovascular diseases, *MET* Metabolic equivalent task, *MVPA* Moderate-to-vigorous physical activity

**Fig. 2:**
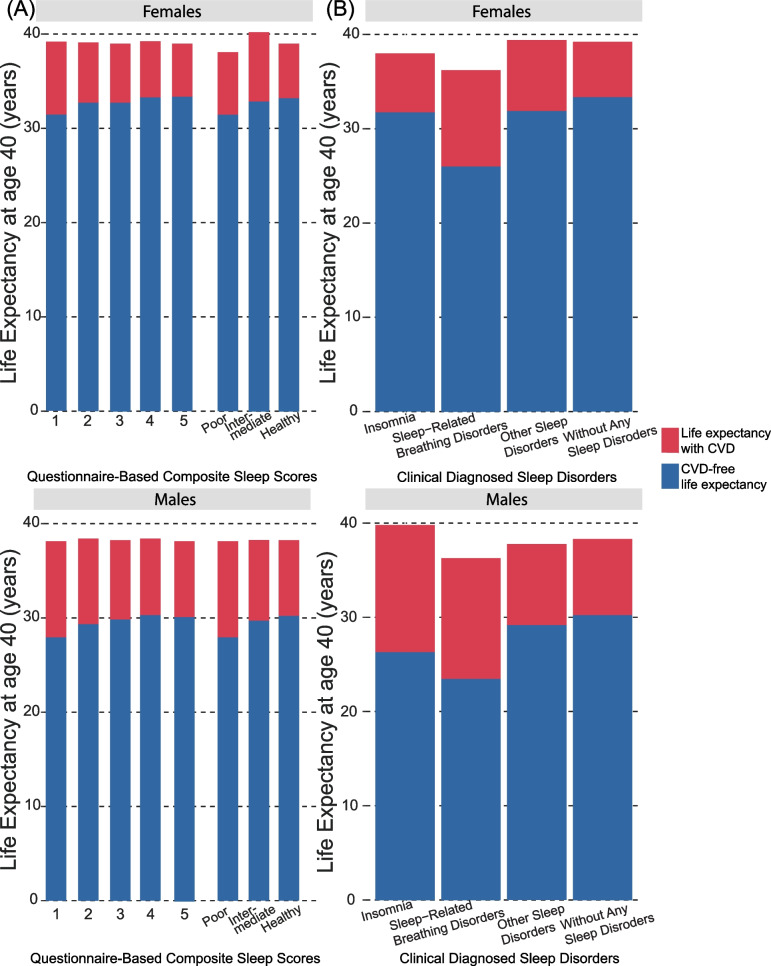
Total and cardiovascular disease-free life expectancy at age 40 among participants with different **A** questionnaire-based composite sleep scores or **B** diagnosed sleep disorders. The results were adjusted for age, socioeconomic status, mental health issues, perceived health, body mass index (BMI), economic activity and shift work, cigarette smoking, alcohol consumption, diet quality, discretionary screen time, and physical activity. Full estimations of each life expectancy were provided in Additional file 1: Table S10-11

**Fig. 3:**
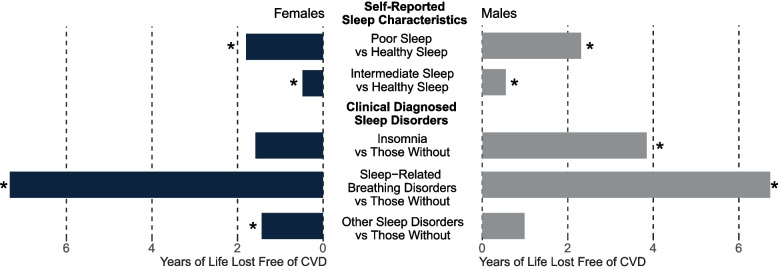
The years of life lost free of cardiovascular disease among participants with questionnaire-based unhealthy sleep or diagnosed sleep disorders. The results were adjusted forage, socioeconomic status, mental health issues, perceived health, body mass index (BMI), economic activity and shift work, cigarette smoking, alcohol consumption, diet quality, discretionary screen time, and physical activity. An asterisk denotes a statistical significance. See Table 2 for exact effect size and confidence intervals; full estimations of each life expectancy are provided in the Additional file 1: Table S10-11

At age 40, total life expectancy was an additional 39.07 [38.95–39.17] and 38.29 [38.16–38.41] years (Additional file 1: Table S10), while CVD-free life expectancy was 33.05 [32.91–33.21] and 30.03 [29.88–30.20] years for females and males (Table 2). Although total life expectancy did not differ between sleep groups (Fig. 2A, Additional file 1: Table S11), there was a gradual decrease in CVD-free life expectancy toward a higher number of self-reported sleep characteristics in both sexes (Table 2, Fig. 2A). Women with poor sleep had a CVD-free life expectancy of 31.46 [30.36–32.48] years, while those with healthy sleep had 33.26 [33.08–33.46] years (difference: 1.80 [0.96–2.75]). The Women’s intermediate group lost 0.48 [0.41–0.55] life years (Table 2, Fig. 3). A similar pattern emerged among males, yet more pronounced than among women. Poorly sleeping men expected a 27.96 [26.80–29.02] CVD-free life years, and healthy sleepers had 30.27 [30.07–30.49] years (difference: 2.31 [1.46–3.29]). The composite sleep categories suggested joint effects of different sleep characteristics compared to models only focusing on a single characteristic (Table 2, Additional file 1: Fig. S4). The corresponding HR for CVD were 1.13 [1.05–1.22] and 1.17 [1.09–1.26] in poor sleepers compared to healthy ones among females and males (Additional file 1: Fig. S5).

**Table 2:** Cardiovascular-free life expectancy and years of life lost at age 40 among participants with different sleep exposures

	Females		Males	
Exposures	CVD-free life expectancy	CVD-free years of life lost	CVD-free life expectancy	CVD-free years of life lost
Total included participants	33.05 [32.91–33.21]		30.03 [29.88–30.20]	
Primary care subsample	33.37 [33.16–33.60]		30.24 [30.01–30.50]	
Sleep characteristic^a^				
Usual insomnia complaints	32.98 [32.67–33.28]	**0.30 [0.18–0.43]**	30.09 [29.66–30.50]	0.14 [− 0.05–0.35]
No usual insomnia complaints	33.28 [33.07–33.47]		30.23 [30.01–30.45]	
Inadequate sleep duration	32.63 [32.32–32.93]	**0.63 [0.51–0.76]**	29.86 [29.50–30.21]	**0.32 [0.19–0.45]**
Adequate duration	33.26 [33.05–33.45]		30.18 [29.94–30.42]	
Snoring	33.07 [32.76–33.37]	− 0.01 [− 0.08–0.06]	30.06 [29.80–30.32]	0.09 [0.00–0.17]
No snoring	33.26 [33.06–33.45]		30.41 [30.16–30.66]	
Evening chronotype	33.21 [32.93–33.47]	**0.19 [0.07–0.32]**	30.20 [29.90–30.51]	**0.35 [0.31–0.4]**
Morning chronotype	33.20 [32.99–33.39]		30.29 [30.05–30.52]	
Frequent daytime sleepiness	32.33 [31.00–33.37]	**0.92 [0.04–2.06]**	29.85 [28.40–30.98]	0.41 [− 0.55–1.66]
Infrequent daytime sleepiness	33.24 [33.06–33.41]		30.26 [30.05–30.46]	
Composite sleep score^b^				
≤ 1	31.46 [30.36–32.48]	**1.90 [1.10–2.76]**	27.96 [26.80–29.02]	**2.21 [1.48–3.07]**
2	32.73 [32.27–33.19]	**0.62 [0.41–0.85]**	29.36 [28.86–29.86]	**0.82 [0.61–1.02]**
3	32.78 [32.51–33.09]	**0.57 [0.5–0.65]**	29.84 [29.57–30.15]	**0.33 [0.21–0.45]**
4	33.32 [33.09–33.58]	0.04 [− 0.03–0.11]	30.30 [30.06–30.57]	− 0.13 [− 0.25–0.01]
5	33.36 [33.07–33.68]		30.17 [29.81–30.56]	
Poor (≤ 1)	31.46 [30.36–32.48]	**1.80 [0.96–2.75]**	27.96 [26.80–29.02]	**2.31 [1.46–3.29]**
Intermediate (2 to 3)	32.77 [32.54–33.03]	**0.48 [0.41–0.55]**	29.72 [29.48–29.97]	**0.55 [0.49–0.61]**
Healthy (≥ 4)	33.26 [33.08–33.46]		30.27 [30.07–30.49]	
Clinical diagnosed sleep disorder^a^				
Insomnia	31.77 [28.23–33.78]	1.58 [− 0.24–4.97]	26.32 [21.34–29.73]	**3.84 [0.61–8.59]**
Without this condition	33.36 [33.11–33.57]		30.16 [29.92–30.39]	
Sleep-related breathing disorders	26.01 [22.80–28.21]	**7.32 [5.33–10.34]**	23.48 [21.54–25.10]	**6.73 [5.31–8.48]**
Without this condition	33.34 [33.09–33.56]		30.21 [29.96–30.44]	
Other sleep disorders	31.91 [29.81–33.34]	**1.43 [0.20–3.29]**	29.19 [27.11–30.77]	0.99 [− 0.36–2.85]
Without this condition	33.33 [33.09–33.55]		30.18 [29.93–30.41]	
With any sleep disorders	30.64 [29.46–31.74]	**2.73 [1.85–3.72]**	26.46 [25.30–27.58]	**3.79 [2.89–4.71]**
Without any sleep disorders	33.37 [33.16–33.60]		30.24 [30.01–30.50]	

Data were shown in estimation [95% CI], with values in bold denoting statistical significance. For all the estimations, we applied median or mean value for categorical or numerous covariates, respectively, and for estimations of each self-reported sleep characteristic or each diagnosed sleep disorder, the remaining characteristics or disorders were set to healthy

*Abbreviations*: *CVD* Cardiovascular diseases

^a^Compared to those without poor sleep characteristics/diagnosed conditions. ^b^Compared to the healthiest group (five score/healthy group). Full estimations of each life expectancy were provided in Additional file 1 pp 14

Among the 140,181 participants with primary care data available, 1203 had an insomnia diagnosis in the last 2 years before their enrollment, while 2441 and 6489 were diagnosed with sleep-related breathing disorders and other sleep disorders (Additional file 1: Table S9). A confusion matrix confirmed an assured negative predictive value of self-reported with diagnoses (Additional file 1: Table S12). During an average follow-up of 11.8 (1.2) years and 1,655,597 person-years at risk of death, 5996 died, and 23,480 developed CVD. Total life expectancy was an additional 39.22 [39.05–39.37] and 38.29 [38.09–38.47] years (Fig. 2B, Additional file 1: Table S10), while CVD-free life expectancies were 33.37 [33.16–33.60] and 30.24 [30.01–30.50] years for females and males (Table 2, Fig. 2B). Contrary to self-report sleep, participants with any clinical sleep disorders lost up to 1.16 [0.43–2.21] life compared to those without (Additional file 1: Table S10). Those diagnosed with sleep disorders showed similar characteristics to those with poor self-reported sleep (Additional file 1: Table S9). Women diagnosed with sleep-related breathing disorders or other sleep disorders lost 7.32 [5.33–10.34] (HR: 2.09 [1.87–2.35]) or 1.43 [0.2–3.29] (HR: 1.20 [1.08–1.34]) CVD-free years of life compared to those without the corresponding diagnosis, respectively (Table 2, Fig. 3, Additional file 1: Fig. S6). In men, those with insomnia or sleep-related breathing disorders expected a significant CVD-free life loss by 3.84 [0.61–8.59] (HR: 1.49 [1.23–1.80]) or 6.73 [5.31–8.48] (HR: 1.83 [1.68–1.99]) years, respectively. Complete estimations of life expectancy and HRs were provided in the Additional file 1: Table S1 and Fig. S5-6.

## Discussion

To our knowledge, the current study provides the first comprehensive analyses of sleep measures with life expectancy, utilizing cohort study data and primary care electronic health records to identify sleep disorders. At 40 years of age, participants with self-reported poor sleep lost 1.8 and 2.3 years of life free of CVD in women and men, respectively, compared to those who reported healthy sleep. The diagnosis of sleep-related breathing disorders was associated with the largest loss of life years without diseases in both sexes, up to 7.3 years, while diagnosed insomnia compromised life years in men by 3.8 years. Our findings strongly suggest that people with either self-reported or diagnosed unhealthy sleep have less life expectancy free of CVD.

“Seven to 9 h of good-quality sleep on a regular basis, with consistent bed and wake-up times” is the only national (Canada) guideline on sleep [3], based on a review of reviews on duration and a de novo systematic review of the regularity/consistency and chronotype/timing [5, 6]. Although we could not address sleep regularity/consistency as it was outside the scope of the UK Biobank questionnaires, aligned with this guideline, our finding supported that sleep duration and chronotype impact CVD-free life expectancy in both sexes. However, “sleep quality” is a poorly defined term encompassing various measures and characteristics, so in the present study, we applied available CVD risk factors, including insomnia complaints, snoring, and daytime sleepiness. Despite the controversial outcomes between recent systematic reviews of insomnia [25, 26], our analyses showed that insomnia impacted CVD-free life expectancy differently between sexes, as highlighted by the distinct effects of self-reported complaints and clinical insomnia. Although we found no association of self-reported/co-sleeper-reported snoring with life expectancy outcomes, clinical sleep-related breathing disorders were found to compromise CVD-free life expectancy in both sexes by up to 7.3 years. Our results are consistent with those from the only existing review on topic [26] and suggest that women with frequent daytime sleepiness impacted the number of CVD-free years. The absence of clinical diagnosis of excessive daytime sleepiness and the low prevalence (i.e., 33 out of 140,181 participants) of circadian rhythm sleep disorders, the corresponding clinical diagnosis of chronotype, prohibited us from further investigating objective measurements.

Besides the independent effect of each characteristic, composite sleep measures provide more comprehensive insights and a more coherent public health message [3] and are increasingly used in cohort studies [8, 9]. Although there is still no systematic review available on composite sleep exposures, the present results, consistent with existing individual studies [8, 9], suggested that people with multiple poor sleep characteristics had a shorter CVD-free year in both sexes. Our study further suggested that this association is more prominent in males, while the very few longitudinal results of sleep and health examining sex as an effect modifier are incongruous [6, 27–29]. The systematic review conducted by He et al. (2020) found that the mortality risk of long sleep duration was higher in females, but the mortality risk of short sleep was higher in males [27]. However, the systematic review of the Canadian national guideline and recent prospective studies found no sex differences in the mortality and CVD risks of non-adequate sleep duration [6, 28, 29]. Since capacities of sleep measurements vary between cohorts, arbitrariness in the included sleep measures and the subsequent adjustments hamper the aggregation of findings. More studies with detailed outcomes on each sleep measure are warranted to facilitate future harmonization and consensus on essential sleep characteristics selections.

This study’s main strength and novelty is the use of primary care data linkage to identify sleep disorders, allowing us to compare the differences between clinical sleep disorders and self-reported poor sleep with detailed sleep questionnaires. The large sample size further allows us to explore effect size with adequate statistical power. National hospitalization and death records across all participants reduce the potential measurement bias of outcomes and allow us to perform life expectancy analyses comprising morbidity and mortality. However, our study has several limitations. First, sleep-related clinical events other than insomnia and sleep-related breathing disorders were rare and thereby had to be grouped, compromising the specificity of our analyses. Second, the UK Biobank has poor representativeness by oversampling the high socioeconomic status, while our exclusion criteria further oversampled the healthy population (Additional file 1: Table S7), so our findings might not be generalizable to a population with different socioeconomic status. The low prevalence of clinical sleep disorders also indicated a potential underestimation due to the undiagnosed. However, recent UK Biobank evidence suggests that poor representativeness is unlikely to influence the associations between lifestyle exposures and mortality [30]. Third, we could not rule out immortal-time bias as the current analyses only followed up mid-age healthy adults. Fourth, we could not distinguish CVD recovery and relapse events based on available data, so we could not avoid overestimating the time spent on diseases. Fifth, we only considered baseline measurements of exposures and covariates to maintain statistical power, although our previous finding suggested a reasonable temporal consistency [8]. Lastly, in the current study, we conservatively chose underlying conditions as covariables since comorbidities could be on the causal pathway (i.e*.*, mediators). We could not eliminate the potential residual confounding (Additional file 1: Fig. S3), so the true causality could be biased.

## Conclusions

In summary, this study provides further support for the advantage of a composite sleep score based on easily applied self-reported questions. With access to primary care records, our findings shed light on how clinical sleep disorders could be adversely associated with CVD-free life expectancy. These results could lay the cornerstone for future research on early prevention against poor sleep and improving CVD-free life expectancy among poor sleepers. Especially, we hope that the current outcomes can serve as a reference for policymakers and professionals to develop national sleep guidelines.

## Supplementary Information

**Additional file 1: Method S1.** Detailed Start and Censor for Each Health-Related Data Linkage. **Method S2.** Primary Care Data Linkage, Cleaning, and Harmonization. **Table S1.** Definitions of Cardiovascular Disease. **Table S2.** Original Questions for Self-Reported Sleep Characteristics. **Table S3.** Definitions of Clinical Sleep Disorders in ICD and OPCS. **Table S4.** Definitions of Clinical Sleep Disorders in CTV3. **Table S5.** Definition of Sleep Pill Prescription in BNF. **Table S6.** Original Questions and Definitions for Covariates. **Table S7.** Comparison Sample Characteristics between Participants Excluded and Included. **Table S8.** Basic Characteristics of Participants with Different Self-Reported Sleep Characteristics. **Table S9.** Basic Characteristics of Participants with Different Diagnosed Sleep Disorders. **Table S10.** Detailed Total, CVD-Free, with CVD Life Expectancy Estimation at Age 40. **Table S11.** Total Life Expectancy and Years of Life Lost at Age 40 among Participants with Different Sleep Exposures. **Table S12.** Confusion Matrix of Self-Reported Insomnia/Snoring with Diagnosed Insomnia/Sleep-Related Breathing Disorders. **Figure S1.** Primary Care Clinical Events Data Flowchart. **Figure S2.** Primary Care Prescription Data Flowchart. **Figure S3.** a priori Defined Directed Acyclic Graphs. **Figure S4.** Total and Cardiovascular Disease-Free Life Expectancy at Age 40 among Participants with Self-Reported Sleep Characteristics. **Figure S5.** Risks for Cardiovascular Diseases between Participants with Different Self-Reported Sleep Characteristic. **Figure S6.** Risks for Cardiovascular Diseases between Participants with Different Diagnosed Sleep Disorders.

## Data Availability

The data that support the findings of this study are available from the UK Biobank but restrictions apply to the availability of these data, which were used under license for the current study, and so are not publicly available. Data are however available from the authors upon reasonable request and with permission of the UK Biobank.

## Abbreviations

BMI: Body mass index
BNF: British National Formulary
CTV3: Clinical Terms Version 3
CVD: Cardiovascular disease
dm + d: Dictionary of Medicines and Devices
HR: Hazard ratios
ICD: International Classification of Diseases
NHS: National Health Service
Read v2: Read codes version 2
